# Streptococcus invasive locus (sil) in Group A Streptococcus causing non-invasive infections in Chennai, South India

**DOI:** 10.1186/1471-2334-12-S1-P62

**Published:** 2012-05-04

**Authors:** B Divya Bajoria, Thangam Menon

**Affiliations:** 1 Department of Microbiology, Dr. A.L.M Post Graduate Institute of Basic Medical Sciences, University of Madras, Taramani, Chennai - 600 113, India

## Introduction

Group A *Streptococcus* (GAS) causes diseases ranging from superficial skin & throat infections to severe life-threatening diseases. *Streptococcus* invasive locus (*sil*) is a virulence gene responsible for pathogenesis of GAS. This study intends to detect the presence of *sil* gene among the non-invasive GAS and its association with toxin genes, erythromycin resistance genes, and *emm* types.

## Methods

A total of 85 GAS isolates (43 pyoderma, 18 pharyngotonsilitis, 24 carrier) were screened for presence of *sil* C&D gene, toxin genes (*spe*A, *spe*B, *spe*C, *spe*G, *sme*Z, *spe*H, *spe*J, *ssa*, *spe*F), and erm genes by PCR. *emm* typing was done by *emm* gene amplification and sequencing.

## Results

Among 85 isolates, 20/85 (23.5%) were positive for *sil* C and 27/85 (31.8%) isolates were positive for *sil* D. Both *sil* C*&*D were present in 20/85 (23.5%) isolates, whereas 58/85 (68.2%) isolates were negative for both *sil* C&D. Comparing the presence of *sil* C&D among the isolates from different sources, no significant difference (p>0.05) was found. There was no significant differences between the toxin gene profile and presence of *erm* genes between *sil-*positive/negative isolates (p>0.05). Thirty nine different *emm* types were observed among the 85 GAS, reflecting a diversity of 45.88%. *emm* types harbouring *sil* were *emm*89.0b(5), *emm*82.1(3), *emm*74.0(2), *emm*80.0(1), *emm*95.0(1), *emm*105.0(1), *emm*11.0(1), *emm*44.0(1), *emm*55.0(1), *emm*66.0(1), *st*2460.1(1), *st*6735.0(1), *st*G652.0(1). Many of these *emm* types were also found among the *sil-*negative strains.

## Conclusion

23.5% of the non-invasive GAS harboured *sil*. There was no specific association of *sil* genes with toxin genes *erm* genes, *emm* types or source of isolation.

